# Investigation of the relationship between red blood cell distribution width and mortality in patients with hemophagocytic lymphohistiocytosis: a retrospective study

**DOI:** 10.1590/1516-3180.2022.0190.R1.17102022

**Published:** 2023-01-06

**Authors:** Chunyan Chen, Shili Zhong, Zhengbin Wu, Hao Tang, Zhen Wang, Dongpo Jiang

**Affiliations:** I MD, MSc. Physician, Department of Intensive Care Unit, Daping Hospital, Army Medical University, Chongqing, China.; II MD, MSc. Physician, Department of Intensive Care Unit, Daping Hospital, Army Medical University, Chongqing, China.; III MD, MSc. Physician, Department of Intensive Care Unit, Daping Hospital, Army Medical University, Chongqing, China.; IV MD, PhD. Assistant Professor, Department of Intensive Care Unit, Daping Hospital, Army Medical University, Chongqing, China.; V MD, PhD. Assistant Professor, Department of Intensive Care Unit, Daping Hospital, Army Medical University, Chongqing, China.; VI MD, MSc. Physician, Department of Intensive Care Unit, Daping Hospital, Army Medical University, Chongqing, China.

**Keywords:** Lymphohistiocytosis, hemophagocytic, Mortality, Erythrocytes, Erythrocyte count, Erythrocyte indices, Inflammation, Red blood cell distribution width, Prediction, Correlation

## Abstract

**BACKGROUND:**

Red blood cell distribution width (RDW) is related to sepsis-related mortality. Hemophagocytic lymphohistiocytosis (HLH) is a syndrome caused by severe infection, tumors, or autoimmunity without a specific diagnosis.

**OBJECTIVE:**

To explore the correlation between RDW and mortality in patients with HLH.

**DESIGN AND SETTING:**

A retrospective study conducted in a hospital in China.

**METHODS:**

A total of 101 inpatients with HLH from January 1, 2017 to December 31, 2021 were divided into non-survivor (n = 52) and survivor (n = 49) groups. A non-parametric test was used to analyze demographic, clinical, and laboratory data between groups. Independent variables with P < 0.05 were analyzed using binary logistic regression to screen out mortality-related variables. Selected variables were subjected to multivariate logistic regression analysis, and those with strong correlations were screened. Receiver operating characteristic (ROC) curves of strongly correlated variables and area under curve (AUC) values were obtained.

**RESULTS:**

The APACHE II score, RDW, and platelet (PLT) and fibrinogen (FIB) levels (P < 0.05) different significantly. RDW, PLT, FIB were correlated with mortality. The AUC values of RDW, PLT, and FIB were 0.857, 0.797, and 0.726, respectively. RDW was associated with mortality in patients with HLH (P < 0.01, cut-off value: 16.9). The sensitivity and specificity of predicting mortality were 97.96% and 96.1%, respectively.

**CONCLUSION:**

Logistic regression analysis showed a correlation between RDW and patients’ mortality. Therefore, RDW can be used to predict mortality in patients with HLH.

## INTRODUCTION

Hemophagocytic lymphohistiocytosis (HLH) is a high inflammatory response syndrome, wherein uncontrolled immune activation leads to multiple organ failure, with high mortality.^1,2^ Impaired immune function, such as that of natural killer (NK) or T cells, is a key factor in the occurrence of HLH.^1,3^ Overactivation of macrophages can induce hemophagocytosis and a cytokine storm, resulting in clinical manifestations, such as fever, enlargement of the liver and spleen, and reduction of extracellular cells.^4,5^

The concept of HLH was proposed by two pediatricians, Scott and Robb Smith, in 1939.^6^ Therefore, our understanding of HLH was initially concentrated in children, and adult HLH was gradually recognized.^7^ Adult HLH in Italy, Sweden, and the United States have an annual incidence rate of 1 per 800,000 people.^8,9^ Increasing annual infections, tumors, and autoimmune diseases are the leading causes of secondary adult HLH in China.^10,11^

Red blood cell distribution width (RDW), derived from whole blood count, is a parameter reflecting the volume heterogeneity of red blood cells that can classify anemia.^12,13^ Elevated RDW is considered an inflammatory marker that can predict the adverse prognosis of various diseases, including heart failure, acute renal injury, sepsis, and cancer. Platelets play an important role in regulating inflammation and innate immunity.^13,14^ They adhere to endothelial cells during acute inflammation, mediating neutrophil chemotaxis, infiltration, and secretion of pro-inflammatory chemokines. Severe infection can lead to a decreased platelet count. Studies^15,16^ have shown that platelet count is a predictor of mortality. Adult secondary HLH is associated with rapid progress and high mortality. More biological indicators are needed to predict patients’ mortality as they have attracted clinicians’ attention and improved patient vitality thus far.

RDW is also considered a novel inflammatory predictor in various conditions including functional bowel conditions,^17^ autoimmune diseases,^18^ rheumatoid arthritis,^19^ degenerative vertebral conditions,^20^ malignancy,^21^ autoimmune hepatitis,^22^ and even coronavirus disease 2019 infection.^23^ Moreover, increased RDW has been linked with multiple hospital admissions in patients with chronic conditions.^24^ Since RDW and cardiovascular conditions are associated with inflammation, RDW could also be associated with HLH prognosis.

## OBJECTIVE

This study aimed to explore the correlation between RDW and mortality in patients with HLH.

## METHODS

### Ethics committee approval

This study was approved by the ethics committee of Daping Hospital of Army Medical University (Approval No. 2022-11; January 24, 2022). It was performed in accordance with the Helsinki Declaration of 1975, as revised in 2013 (http://www.wma.net/en/20activities/10ethics/10helsinki/).

### Patients

**Figure 1.**
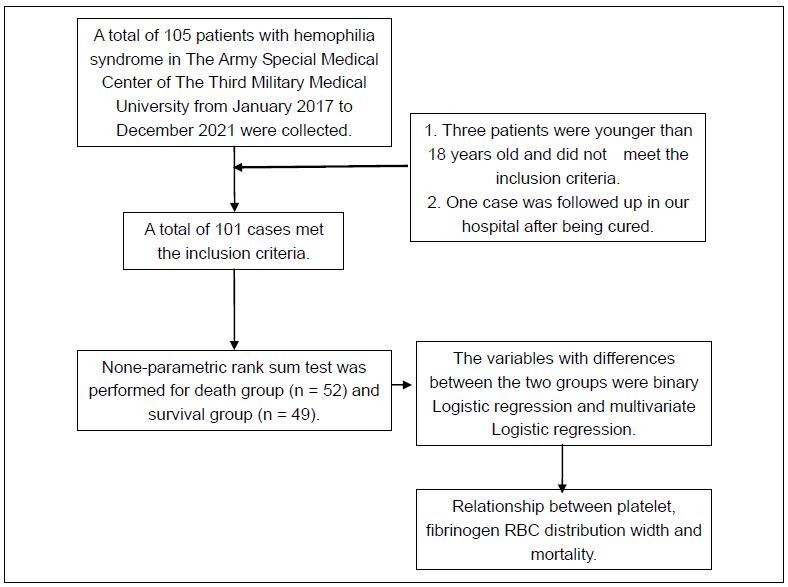
Flow chart for the study selection process.

This retrospective study was conducted among 105 inpatients with HLH from January 1, 2017 to December 31, 2021. According to the inclusion criteria, of these patients, three who were younger than 18 years and one with recurrence after treatment were excluded. Finally, a total of 101 patients were included in this study (Figure 1). These patients were divided into non-survivor (n = 52) and survivor (n = 49) groups.

### Inclusion criteria

The patients involved in this study had to comply with the following requirements. First, the patient must be aged over 18 years. Second, the patient must meet the following criteria (HLH-2004 diagnostic criteria):^25^ 1) fever ≥ 38.5 ℃; 2) splenomegaly; 3) cytopenia that affects at least two peripheral blood samples of three cell lines (i.e., hemoglobin < 90 g/L, platelet count <100×10^9^/L, and neutrophils <1×10^9^/L); 4) high serum triglyceride (3 mmol/L) and/or low fibrinogen (FIB ≤ 1.5 g/L) levels; 5) hemophagocytosis of the bone marrow, spleen, or lymph nodes; 6) low or absent NK cell activity; 7) ferritin ≥ 500 μg/L; and 8) soluble CD25 (soluble interleukin-2 [IL-2] receptor) ≥ 2400 U/mL.

### Parameter measurement

The non-rank sum test was used to analyze the following parameters of the two groups: patient age, sex, Acute Physiology and Chronic Health Evaluation II (APACHE II) score, APACHE II death risk coefficient, sequential organ failure assessment (SOFA) score, blood routine, coagulation, liver and renal function, interleukin (IL)-2, IL-6, ferritin, bone marrow puncture, immunoglobulin, and hormone use. Moreover, this study also measured whether the liver and kidney were enlarged.

### Statistical analysis

In this study, data were analyzed using the STATA statistical software (Corp, College Station, Texas, United States). Kolmogorov–Smirnov test was used to verify whether all data conform to the normal distribution. The measurement data satisfying the normal distribution were represented as means ± standard deviations. The measurement data that did not meet the normal distribution were represented as medians (P25, P75). Categorical variables were expressed as percentages (%). Two groups of data were analyzed using a non-parametric test. The assumption of normality of the variances required for comparison was analyzed with the Kolmogorov–Smirnov test. P < 0.05 showed a statistical difference. The variables with statistical differences were used as independent variables and death as the dependent variable for binary and multiple logistic regression analyses (P < 0.05 indicated the correlation). Finally, a Cox proportional hazards model was established for the relevant variables to generate a receiver operating characteristic (ROC) curve that can improve the prediction accuracy of RDW and platelet and FIB levels and calculate the area under the curve (AUC).

## RESULTS

### Comparison of clinical data and laboratory records

In this study, the demographics, clinical data, and laboratory results of patients with HLH in the non-survivor and survivor groups were analyzed and compared (Table 1). The results showed no significant difference between non-survivor and survivor groups regarding population data, including age and sex (P > 0.05). There were significant differences in most clinical data and laboratory indicators between both groups, including APACHE II and SOFA scores, APACHE II death risk coefficient, levels of leukocytes, hemoglobin, high-sensitivity C-reactive proteins, platelets, IL-6, FIB, D-dimer, albumin, globulin, γ-glutamyl transpeptidase, lactate dehydrogenase, total cholesterol, and blood lactic acid; percentage of neutrophils; lymphocyte count; RDW; international normalized ratio; activated partial thromboplastin time; oxygenation index; length of hospital stay; hormone treatment; and proportion of tumor diseases. Bone marrow puncture results showed that phagocytes and liver size increased (Table 1, all P < 0.05). However, there was no significant difference between non-survivor and survivor groups in other parameters and data listed in Table 1 (all P > 0.05).

**Table 1.** Demographics, clinical data, and laboratory findings of patients with hemophagocytic lymphohistiocytosis (HLH) in the non-survivor and survivor groups

Characters	Non-survivor (n = 52)	Survivor (n = 49)	P value (P < 0.05)
Age (years), Mean (Min, Max)	50.8 (13.0, 89.0)	48.7 (14.0, 75.0)	0.67
Male/Total (%)	38%	42%	0.28
APACHEII SCROE, Mean (Min, Max)	29.4 (15.0, 47.0)	20.9 (8, 31)	**< 0.001**
APAHCHEII Dead Rate, Mean (Min, Max)	71.9 (32.6, 97.9)	51.8 (16.8, 88.3)	**< 0.001**
SOFA, Mean (Min, Max)	10.6 (3.0, 18.0)	6.8 (2, 14)	**< 0.001**
Temperature upon admission, Mean (Min, Max)	39.6 (37.0, 41.2)	39.4 (36.9, 41)	0.314
WBC (10^12^/L), Mean (Min, Max)	0.9 (0.1, 189.3)	2.7 (0.8, 20.0)	0.05
HB (g/L), Mean (Min, Max)	63 (42, 95)	71 (30, 136)	**0.04**
NEUT (%), Mean (Min, Max)	1.9 (0, 45.2)	1.5 (0, 13.5)	0.38
LY (10^9^/L), Mean (Min, Max)	0.5 (0, 8.2)	0.4 (0.3,1.2)	**0.04**
CRP (mg/L), Mean (Min, Max)	127.0 (8.3, 294.8)	89.9 (0.5, 255.7)	**0.006**
RDW (%), Mean (Min, Max)	20.6 (12.4, 29.4)	16.0 (12.4, 25.1)	**< 0.001**
PLT (10^12^/L), Mean (Min, Max)	23.7 (1.0, 128.0)	51.5 (5, 365)	**< 0.001**
PCT (ng/L), Mean (Min, Max)	9.6 (0.2, 64.5)	3.8 (0.1, 36.3)	0.07
IL-2 (U/mL), Mean (Min, Max)	6,241.1 (48.5, 7500.0)	5,696 (883.0, 7,500.0)	0.33
IL-6 (pg/mL), Mean (Min, Max)	824.5 (1.5, 5000.0)	163.8 (1.5, 1,921.0)	**< 0.001**
INR, Mean (Min, Max)	2.0 (0.6, 16)	1.4 (0.7, 11.5)	**< 0.001**
FIB (g/L), Mean (Min, Max)	1.1 (0.2, 3.6)	2.2 (0.34, 9.7)	**< 0.001**
APTT (s), Mean (Min, Max)	53 (1.4, 240.0)	44.9 (23.6, 240)	**0.01**
DD (μg/L), Mean (Min, Max)	10,308.5 (437.8, 8,6287.0)	4,852.4 (10.6, 48,745)	**0.01**
ALB (g/L), Mean (Min, Max)	22.7 (10.1, 45.6)	24.2 (15, 39.8)	**0.01**
GLOB (g/L), Mean (Min, Max)	22.4 (10.8, 48.0)	25.6 (11.2, 46.8)	**0.01**
AST (U/L), Mean (Min, Max)	671.9 (11.6, 9,315.6)	324.7 (23.6, 2915)	0.87
ALT (U/L), Mean (Min, Max)	545.1 (9.9, 15,876)	234.9 (15.3, 1102.8)	0.26
AKP (U/L), Mean (Min, Max)	297.3 (51.9, 1,091.0)	382.1 (61.9, 1496.2)	0.29
γ-GT (U/L), Mean (Min, Max)	141.4 (21.7, 453.0)	296.1 (11.2, 1320)	**0.04**
LDH (U/L), Mean (Min, Max)	2,415.0 (4.6, 21,205.0)	1,182.8 (78.5, 8,849)	0.05
TB (μmol/L), Mean (Min, Max)	64.5 (4.5, 391.9)	52.5 (6.7, 385)	0.63
DB (μmol/L), Mean (Min, Max)	35.5 (2.0, 336.9)	24.7 (1.5, 231.3)	0.91
IB (μmol/L), Mean (Min, Max)	27.8 (2.9, 160.3)	23.6 (2, 119)	0.93
CHOL (mmol/L), Mean (Min, Max)	3.6 (0, 35.3)	4.8 (1.7, 44.4)	**0.01**
Triglyceride (mmol/L), Mean (Min, Max)	2.9 (0, 16.3)	3.3 (0.8, 9.7)	0.21
Ferritin (ng/mL), Mean (Min, Max)	2,515.7 (307.0, 27,406.0)	1,947.7 (79.5, 7,500)	0.81
Na^+^ (mmol/L), Mean (Min, Max)	132.8 (117.0, 184.0)	131.9 (121, 175)	0.18
K^+^ (mmol/L), Mean (Min, Max)	3.5 (2.2, 5.7)	3.4 (2.5, 5.8)	0.76
FiO_2_ (mmHg), Mean (Min, Max)	168.7 (42.0, 350.0)	256 (80, 402)	**< 0.001**
Lac (mmol/L), Mean (Min, Max)	6.5 (1.7, 17.1)	3.4 (1, 15)	**< 0.001**
SCR (μmol/L), Mean (Min, Max)	120.6 (16.8, 586.7)	91.2 (25.9, 426.8)	0.13
Length of stay (days), Mean (Min, Max)	11.0 (1.0, 17.0)	15.5 (1.0, 48.0)	**0.03**
Hormone therapy, (%)	53.8%	79.6%	**0.04**
Immunoglobulin shock therapy, (%)	25%	28.6%	0.18
Tumor disease, (%)	58%	32.6%	**0.03**
Phagocytes in bone marrow biopsy, (%)	78.8%	96.0%	0.05
Liver enlargement, (%)	38.4%	57.0%	0.05
Spleen enlargement, (%)	82.6%	85.7%	0.22
Lung Infection, (%)	59.6%	53%	0.32

Min = minimum; Max = maximum; APACHE II = Acute Physiology and Chronic Health Evaluation II; SOFA = Sequential Organ Failure Assessment; WBC = white blood cell; HB = hemoglobin; NEUT = neutrophils; LY = lymphocyte; CRP = C-reactive protein; RDW = red blood cell distribution width; PLT = platelet; PCT = procalcitonin; IL-2 = interleukin 2; IL-6 = interleukin 6; INR = International Normalized Ratio; FIB = fibrinogen; APTT = activated partial thromboplastin time; DD = D-dimer; ALB = albumin; GLOB = globulin; AST = aspartate aminotransferase; ALT = alanine aminotransferase; AKP = alkaline phosphatase; γ-GT = γ-glutamyl transpeptidase; LDH = lactate dehydrogenase; TB = total bilirubin; DB = direct bilirubin; IB = indirect bilirubin; CHOL = cholesterol; Na^+^ = sodium ion; K^+^ = potassium ion; FiO_2_^+^ = fraction of inspiration O_2_; Lac = lactic acid; SCR = creatinine. The P values in bold font represent significant differences.

### RDW, FIB, and platelets were positively correlated with mortality in patients with HLH

This study analyzed the correlation between RDW and other laboratory parameters (Table 2). The results showed a significant positive correlation between RDW and patients’ mortality (P = 0.01, odds ratio [OR]: 0.97, 95% confidence interval [CI]: 1.31–2.97). FIB (P = 0.05, OR: 0.43, 95% CI: 0.18–1.02) and platelets (P = 0.04, OR: 0.99, 95% CI: 0.95–1.02) were slightly to moderately correlated with patients’ mortality (Table 2). After binary and multiple logistic analyses, the results showed no significant difference between other indexes and patients’ mortality (Table 2, all P > 0.05).

**Table 2.** Correlation analyses between red blood cell distribution width (RDW), fibrinogen and platelets levels, and mortality in patients with hemophagocytic lymphohistiocytosis (HLH)

Variables	Survival rate			
	OR crude	P value	OR adjusted crude (95% CI)	P value
Hemoglobin	0.97 (0.95–0.99)	0.03	0.98 (0.93–1.02)	0.34
C-reactive protein	1.01 (1.00–1.01)	0.01	0.99 (0.99–1.00)	0.44
Red cell distribution width	1.61 (1.31–1.97)	0.00	0.97 (1.31–2.97)	**0.01**
Platelet count	0.98 (0.96–0.99)	0.01	0.99 (0.95–1.02)	**0.04**
Fibrinogen	0.38 (0.23–0.63)	0.00	0.43 (0.18–1.02)	0.05
Globulin	0.95 (0.90–1.00)	0.05	1.06 (0.97–1.15)	0.20
γ-glutamyl transpeptidase	0.99 (0.99-1.00)	0.05	0.99 (0.99–1.00)	0.30

CI = confidence interval; OR = odds ratio; RDW = red blood cell distribution width; HLH = hemophagocytic lymphohistiocytosis. The P values in bold font represent significant differences.

### AUC analyses of RDW, FIB, and platelets for the predictive ability on mortality in patients with HLH

**Figure 2.**
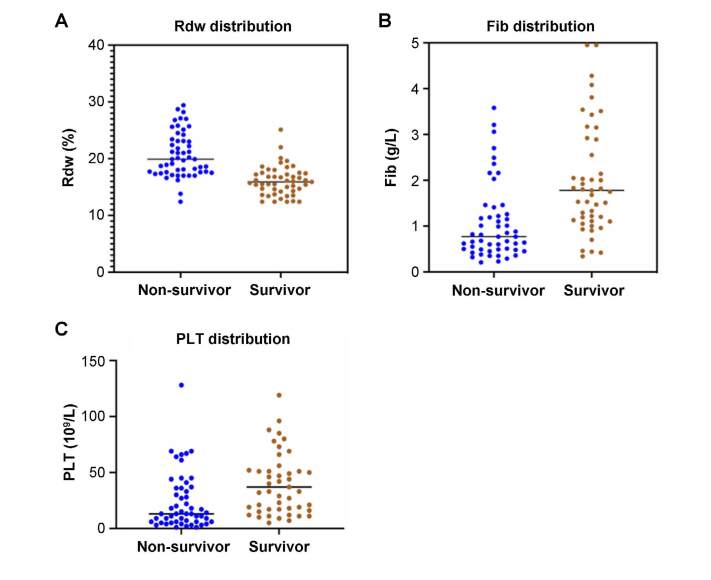
Distribution map of red blood cell distribution width and platelet and fibrinogen levels between non-survivor and survivor groups.

To clarify the predictive ability of RDW, FIB, and platelets on mortality, ROC curves were drawn and analyzed in this study. The AUC curve of RDW was 0.857 (Figure 2A), which was higher than that of FIB (Figure 2B, AUC: 0.726) and platelet (Figure 2B, AUC: 0.797) levels; however, there were no significant differences.

PLT = platelet; Rdw = red blood cell distribution width; Fib = fibrinogen.

### RDW demonstrated higher sensitivity and specificity for predicting mortality in patients with HLH

**Figure 3.**
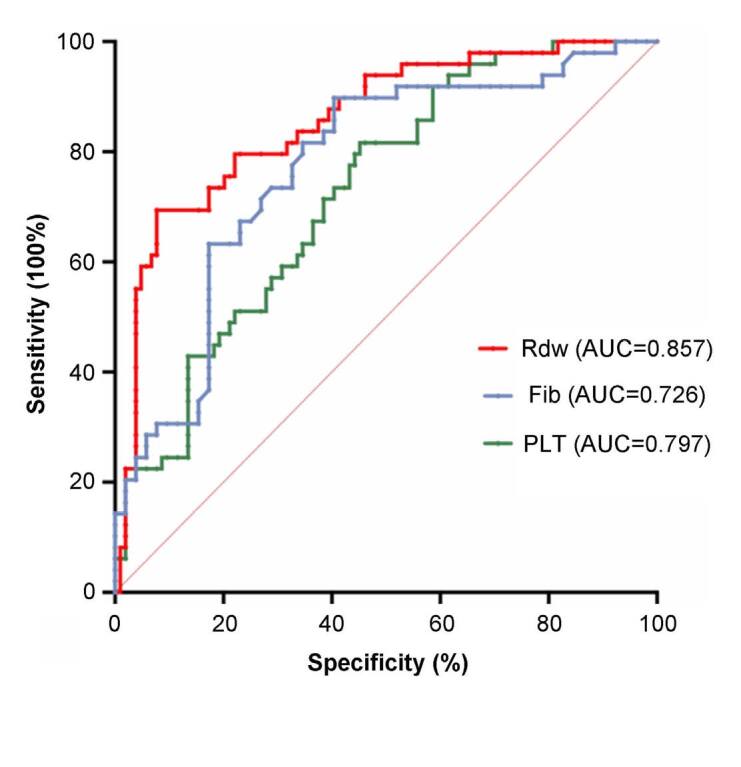
Receiver operating characteristic curves for the red blood cell distribution width and platelet and fibrinogen levels evaluating the area under the curve and predictive value of mortality in patients with hemophagocytic lymphohistiocytosis.

In this study, we assigned the cut-off level as 16.9%. Based on the cut-off value, the sensitivity of RDW for predicting patients’ mortality was 97.96%, whereas the specificity was 96.1% (Figure 3). However, the sensitivity and specificity of FIB were 90.9% and 59.6%, respectively. Therefore, RDW demonstrated higher sensitivity and specificity for predicting mortality in patients with HLH.

PLT = platelet; Rdw = red blood cell distribution width; Fib = fibrinogen; AUC = area under curve.

## DISCUSSION

Adult HLH has received a lot of attention recently, but the current diagnostic criteria have some limitations for clinical patients.^26^ Usually, patients with HLH can only be diagnosed in the middle and late stages of the disease; however, the initial stage is the best treatment period. The RDW is correlated with mortality in patients with sepsis.^12^ Previous studies^27,28^ have reported that RDW is significantly associated with the prognosis of many diseases, such as cancer, sepsis, and cardiovascular disease. However, there are limited reports^29,30^ on the relationship between HLH and RDW. HLH is characterized by an excessive inflammatory response and a cytokine storm. During the inflammatory reaction, proinflammatory cytokines affect the survival of circulating red blood cells, damage the cell membrane of these cells, produce larger and renewed reticulocytes that enter the blood circulation, and increase the distribution width of red blood cells.^31^ This is also a pathophysiological foundation for us to clarify the relationship between HLH and RDW. In this study, most adult HLH cases were induced by infection through blood phagocytosis. Therefore, this study explored whether there was a correlation between RDW and mortality in patients with adult HLH. We found that RDW was positively correlated with mortality in patients with HLH and had a high prediction level. Furthermore, the sensitivity of RDW for predicting mortality was 97.96%, and the specificity was 96.1%, providing more auxiliary diagnostic evidence for patients with HLH.

Hormone pulse therapy is a double-edged sword for clinicians. The application of sufficient hormone pulse at the right time is a rescue treatment for patients, but in the case of severe infection, high-dose hormone pulse may lead to the death of patients. More clinical studies are needed to provide a clinical basis for hormone pulse therapy. In this study, we found significant differences in hormone pulse therapy between non-survivor and survivor groups. We also found that the RDW, and platelets and FIB levels had predictive values for mortality in patients with adult HLH. Fardet et al.^6^ proposed HScore to predict the possibility of a single patient with HLH so that clinicians can make appropriate treatment decisions as soon as possible. However, HScore is a complex index that needs to be improved and comprehensively evaluated after several laboratory tests. Therefore, faster and more easily available laboratory indexes are needed to assist in the diagnosis of adult HLH.^6,7^ The HScore includes the FIB level. This study found that RDW, compared with FIB, demonstrated higher sensitivity and specificity on mortality of patients with HLH (sensitivity: 90.9%, specificity: 59.6%). The sensitivity and specificity of RDW are higher than those of FIB, which has a high predictive value for mortality in adult HLH.

This study had a few limitations. First, this study is a single-center, small sample, cross-sectional retrospective study. Patients with HLH in the survival group were not followed up. Second, IL-2 and ferritin tests in our center have not been analyzed for accuracy. The test results of most patients are greater than a certain value that is not accurate. Therefore, RDW cannot be compared with the predicted values of IL-2 and ferritin.

## CONCLUSIONS

This study collected data from patients with HLH in the hospital and expounded the clinical understanding and treatment perception of HLH from the perspective of the critical care department. This study showed that RDW was associated with mortality in patients with HLH. The cut-off value of RDW was 16.9. The sensitivity and specificity of predicting mortality were 97.96% and 96.1%, respectively. Logistic regression analysis showed a correlation between RDW and mortality. In summary, the RDW can be used as an important index to predict mortality in patients with HLH. The findings of this study suggest that RDW may be suitable as an auxiliary diagnostic method for HLH and an auxiliary means for predicting mortality in adult patients with HLH clinically.
